# Effect of *Laurus nobilis* on bacteria and human transforming growth factor-β1

**DOI:** 10.1590/1806-9282.20230683

**Published:** 2024-04-22

**Authors:** Okan Sancer, Uğur Şahin, Emel Sesli Çetin, Muhammet Yusuf Tepebaşi, Yasemin Cezaroğlu, Göksel Bilir, Sibel Yünlü, Ahmet Koca

**Affiliations:** 1 Suleyman Demirel University, Faculty of Medicine, Department of Medical Biology – Isparta, Turkey.; 2 Suleyman Demirel University, Innovative Technologies Application and Research Center, Genetic Research Unit – Isparta, Turkey.; 3 Suleyman Demirel University, Faculty of Arts and Sciences, Department of Chemistry – Isparta, Turkey.; 4 Suleyman Demirel University, Faculty of Medicine, Department of Medical Microbiology – Isparta, Turkey.; 5 Suleyman Demirel University, Faculty of Medicine, Department of Medical Genetic – Isparta, Turkey.; 6 Isparta University of Applied Sciences, Sütçüler Prof. Dr. Hasan Gürbüz Vocational School – Isparta, Turkey.

**Keywords:** Epicatechin, Laurus nobilis, MTT, o-Coumaric acid

## Abstract

**OBJECTIVE:**

In this study, we aimed to determine the phenolic compounds, the antibacterial activity of extract from *Laurus nobilis* leaves, and its possible effect on transforming growth factor-β1 expression level in peripheral blood mononuclear cells.

**METHODS:**

The phenolic components of *Laurus nobilis* were identified by the high-performance liquid chromatography method. The antibacterial activity of this extract was determined by disk diffusion and broth microdilution methods. The transforming growth factor-β1 expression was analyzed using the RT-qPCR method.

**RESULTS:**

Epicatechin was found in the highest amount and o-coumaric acid in the lowest amount. The half-maximal inhibitory concentration (IC_50_) was determined to be 55.17 μg/mL. The zones of inhibition and minimum inhibitory concentration for *Staphylococcus aureus, Enterococcus faecalis*, and *Klebsiella pneumoniae* were 15, 14, and 8 mm and 125, 250, and 1000 μg/mL, respectively. The change in transforming growth factor-β1 expression levels was found to be statistically significant compared with the control groups (p<0.0001).

**CONCLUSION:**

*Laurus nobilis* extract was found to be effective against bacteria and altered the expression level of transforming growth factor-β1 in peripheral blood mononuclear cells.

## INTRODUCTION

*Laurus nobilis* L. is an aromatic herb that spreads worldwide on the coasts of southern Europe and Asia Minor, where the Mediterranean climate prevails^1^. Laurel is widely used in alternative medicine, food, and cosmetics industries. The main reasons for its use in these areas are due to its antimicrobial, antifungal, antioxidant, anxiolytic, antidepressant, and antistress properties^2^.

Fruits, herbs, and other plant-based foods contain sources of compounds such as (poly)phenols and flavonoids that protect against inflammation and chronic disease. T lymphocytes are instrumental in supporting the production of pro-inflammatory and anti-inflammatory cytokines in tissues and circulation. However, less is known about the relative potency of different (poly)phenols in modulating cytokine release by lymphocytes^3^.

In the immune system, transforming growth factor-β1 (TGF-β1) is a general regulatory activity that affects many types of immune cells. By regulating T lymphocyte development, differentiation, homeostasis, and tolerance, the evolutionarily highly conserved TGF-β cytokine crucially supports a functional T cell pool. TGF-β1 plays a critical role in maintaining peripheral tolerance to endogenous and harmless antigens such as food and commensal bacteria and in controlling the immune response to pathogens^4^.

*Laurus nobilis* is a natural medicinal plant and a rich source of bioactive compounds. The biological properties of its various extracts and its essential oil are documented, in particular their antimicrobial and antioxidant effects^5^ and wound-healing properties in animal models^6^.

Our aim in this study was to determine the effect of the phenolic components of *L. nobilis* leaf extract against the bacteria *Staphylococcus aureus, Enterococcus faecalis,* and *Klebsiella pneumoniae* that cause various infectious diseases and the effect of TGF-β1 expression in human peripheral blood mononuclear cells (PBMCs) in the presence of these bacteria.

## METHODS

**Figure 1:**
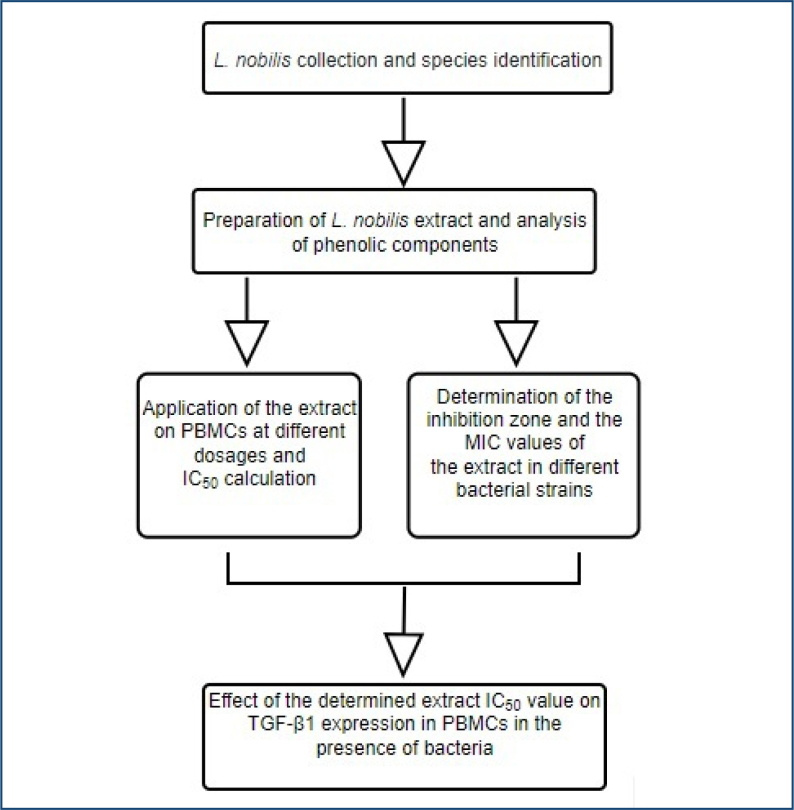
Flowchart of the overall work.

The flowchart of the overall work is shown in Figure 1.

### Plant

Fresh leaves from *L. nobilis* were collected in Isparta/Türkiye in June 2022. The plant material was recorded at the Iğdır University Wildlife Museum (Herbarium Specimen) with reference number INWM00000111. Species verification and taxonomic assessment of *L. nobilis* were completed by Dr. Ahmet KOCA.

### Determination of the phenolic compounds

Determination of phenolic compounds in extracts made by the high-performance liquid chromatography (HPLC) method according to our previous study^7^. After weighing 2.7 g of *L. nobilis* leaves, 30 mL of 99% methanol (Merck, Germany) was added and homogenized with a household mixer. They were mixed in an ultrasonic bath for 1 h. The extracts were filtered on Whatman filter paper (no. 4). Then the filtered extracts were evaporated at 40°C. The remainder in the Erlenmeyer flask was taken with 5 mL of methanol. This solution of 20 μL was injected into the HPLC device. The method of Caponio et al., was used for the determination of phenolic compounds by HPLC^8^. Detection of phenolic compounds was carried out at a wavelength of 278 nm and a flow rate of 0.8 mL/min. A reverse-phase column (5 μm) Agilent Eclipse XDB C18 (4.6 × 250 mm) was used. The column temperature was 30°C. The separation was performed with a binary solvent system using a gradient program. Solution A was 3% acetic acid, and solution B was methanol.

### Preparation of *Laurus nobilis* extract for bacterial and peripheral blood mononuclear cells culture

*Laurus nobilis* extracts were freeze-dried (Labconco FreeZone 6 plus, USA). After the lyophilized samples were dissolved in RPMI 1640 (Biological Industries, Israel) and the extract was filtered with a 0.45-μm filter, the extracts were added to the incubation medium with human peripheral lymphocytes. In parallel, the lyophilized samples were dissolved in dH_2_O and used in bacterial disk diffusion and minimum inhibitory concentration (MIC) tests.

### Isolation of peripheral blood mononuclear cells

Whole blood was collected from lithium heparin tubes from a healthy 34-year-old volunteer who had not been exposed to radiation or drugs for 6 months and who did not smoke.

The isolation of PBMCs was performed according to the protocol of Panda et al.^9^ RPMI 1640 medium and heparinized whole blood samples were mixed 1:1. Then this mixture was slowly added to the tube on Histopaque 1077 (Sigma-Aldrich, Switzerland). The mixture was centrifuged at 2000 rpm for 20 min at 4°C. The “Buffy coat” PBMC layer was removed and transferred to the new tube. After centrifugation, PBMCs and RPMI 1640 medium were resuspended in a 1:1 ratio. Then it was centrifuged at 2500 rpm for 5 min at 4°C. Then the supernatant was taken out. Cell viability was determined to be 98% using trypan blue stain.

### In vitro cell culture

The cell culture of PBMCs was performed according to the protocol of Panda et al^10^. To determine the non-toxic dose of *L. nobilis* leaf extract, 1×10^4^ cells/well PBMCs were seeded at 96-well flat-bottomed microplates (Sarstedt AG, Germany). Then the plate was incubated at 37°C in a 5% CO_2_ and humidified incubator for 24 h. *L. nobilis* extracts of 1000, 500, 250, 125, 62.5, 31.2, 15.6, 7.8, and 0.0 μg/mL were cultured with cells at 37°C in a 5% CO_2_ and humidified incubator for 24 h. The medium consisted of the following components: RPMI-1640 (Biological Industries, Israel) medium supplemented with 10% heat-inactivated fetal bovine serum (Sigma-Aldrich, USA), penicillin (100 IU/mL), and streptomycin (100 μg/mL) (Sigma-Aldrich, USA).

### Determination of IC_50_ value

After incubation, cell viability was evaluated using the 3-(4,5-dimethythiazol-2-yl)-2,5-diphenyltetrazolium bromide (MTT) colorimetric assay (Sigma Aldrich, USA) to determine the IC_50_ value of the *L. nobilis* extract. MTT reagent was added to each well. The final concentration was adjusted to 0.5 mg/mL, and the cells were incubated in a humidified atmosphere at 37°C in 5% CO_2_ for 4 h. The plate was centrifuged at 800 g for 5 min, and then the supernatants were removed. The formazan crystals were dissolved in 200 μLμL of dimethyl sulfoxide (Thermo Fisher Scientific, USA) and shaken at room temperature for 15 min. Optical densities were recorded at 570 nm using a multiscan plate reader (Synergy HTX BioTek, USA)^11^. A plot of viability versus extract concentration was used to calculate IC_50_ values for PBMCs.

### The bacteria strains

*Staphylococcus aureus* (ATCC: 27853), *E. faecalis* (ATCC: 29212), and *K. pneumoniae* (ATCC: 700603) were used in this study. All the strains were obtained from the Microbiology Laboratory at Suleyman Demirel University Hospital, Isparta.

### Antibacterial susceptibility of *Laurus nobilis* leaf extract using the disk diffusion method

The disk diffusion method for antibacterial susceptibility testing was performed using the Kirby-Bauer Disk Diffusion Susceptibility Test Protocol^12^. A 6-mm sterile disk filter paper (Schleicher and Schul, 2668, Dassel, Germany) was applied by impregnating 50 μL of *L. nobilis* leaf extract. The bacterial cultures were inoculated on Nutrient Broth (Becton Dickinson and Company, USA) and incubated at 37°C for 24 h. Adequate quantities of Mueller Hinton Agar (GBL, Türkiye) were dispensed into sterile plates and subjected to solidification under aseptic conditions. Bacterial crop counts were adjusted to yield 1×10^6^ using the McFarland standard count method. The test microorganisms (0.1 mL) were inoculated with a sterile swab on the surface of the suitable plate solid medium. The agar plates inoculated with the test microorganisms were incubated for 1 h before placing the paper disk impregnated with the extract onto the plates. The bacterial plates were incubated at 37°C for 24 h. Following incubation, the parameters of the growth inhibition zones of all plates were measured in millimeters. Gentamicin (10 μg/disk) (Becton Dickinson and Company, USA) and meropenem (10 μg/disk) (Becton Dickinson and Company, USA) for *K. pneumoniae*, penicillin (1 μg/disk) (Becton Dickinson and Company, USA) and cefoxide for *S. aureus* (30 μg/disk) (Becton Dickinson and Company, USA), and ampicillin (2 μg/disk) (Becton Dickinson and Company, USA) and vancomycin (5 μg/disk) (Becton Dickinson and Company, USA) for *E. faecalis* were used as positive control.

### Determination of the minimum inhibitory concentration

The MICs of the raw extracts were achieved by broth micro-dilution using the 96 multiwell microtitration plates^13^. *L. nobilis* extracts (1000 μg/mL) and Mueller Hinton broth (GBL, Türkiye) were applied in the first row of the plate. Mueller Hinton broth was added to other wells. Then, serial dilutions were applied at the rate of 1/2 from the first well to the last well. Standard bacterial strains were adjusted to 0.5 McFarland (10^8^ CFU/mL) turbidity standard and diluted 1/100 with Mueller Hinton Broth (GBL, Türkiye) to 10^6^ CFU/mL. Finally, 10 μL of bacterial suspension was added to each well. The plates were incubated for 24 h at 37°C. The lowest concentration of plant extract that inhibited bacterial growth was considered the MIC.

### Transforming growth factor-β1 expression

Peripheral blood mononuclear cells (1×10^4^ cells/well) were incubated at 37°C in a 5% CO_2_ and humidified incubator for 24 h. Then PBMCs and bacterial strains (10^6^ CFU/mL) were cultured with a determined IC_50_ value, 37°C in 5% CO_2_, and humidified atmosphere for 24 h. Total RNA from PBMCs was extracted with the Hibrigen total RNA isolation kit (Hibrigen, Türkiye). RNA purity and concentration were determined with a NanoDrop ND-1000 spectrophotometer (NanoDrop Technologies, Inc., DE); 1 μg RNA was reverse transcribed with the 5× i-Script RT supermix and nuclease-free water (BioRad, USA). The total volume was adjusted to 20 μL. Primer designs were performed by detecting specific mRNA sequences. Possible primer sequences were tested using the NCBI website. TGF-β1 (Forward 5′-CAATTCCTGGCGATACCTCAG-3′ and Reverse 5′-GCACAACTCCGGTGACATCAA-3′), primers were designed to amplify. The β-actin gene was used as a housekeeping gene and the CT values of this gene were used for normalization. Notably, 0.1 mL PCR tubes were used in the instrument, and the final reaction volume was 20 μL. The reaction mixture was prepared according to the manufacturer's protocol (A.B.T., Turkey). The resulting reaction mixture was loaded into a real-time qPCR instrument with a thermal cycle determined by the kit manufacturer's protocol.

The CT values of the target genes were determined, and formula 2^–ΔΔCt^ (Livak method) was used to determine their relative expression levels^14^.

### Statistical analysis

The results of the TGF-β1 expression study were evaluated using the SPSS 18.0 statistical analysis software (SPSS Inc., USA). Comparisons between groups were performed in the present study by one-way analysis of variance and Tukey analysis. PBMC culture, MTT assay, and TGF-β1 expression were performed in triplicate. Concentration-response curves and IC_50_ values were generated with GraphPad Prism 5.

## RESULTS

### Phenolic compound analysis

In our study, protocatechic acid, p-hydroxybenzoic acid, catechin, luteolin, caffeic acid, camperol, epicatechin, o-coumaric acid, vanillin, ferulic acid, rutin, p-coumaric acid, and cinnamic acid were detected. The analyses of the phenolic compounds of the *L. nobilis* extract are presented in Table 1.

**Table 1:** Analysis result of the phenolic compounds.

Phenolic compounds	Laurus nobilis (μg/g)
Gallic acid	*
Protocatechic acid	85.2
Catechin	173.2
p-Hydroxybenzoic acid	123.6
Chlorogenic acid	*
Caffeic acid	78.2
Epicatechin	2113.7
Syringic acid	*
Vanilin	48.9
p-Coumaric acid	16.7
Ferulic acid	64.2
Sinapinic acid	*
Benzoic acid	*
o-Coumaric acid	2.7
Rutin	246.2
Hesperidin	*
Rosmarinic acid	*
Eriodictiol	*
Cinnamic acid	5.5
Quercetin	*
Luteolin	14.7
Kamferol	34.3

* Could not be detected.

### In vitro viability assay

The IC_50_ of *L. nobilis* extract was determined to be 55.17 μg/mL on PBMCs. The results show that increasing concentrations of *L. nobilis* extract leads to a reduction in the survival rate of PBMCs.

### Antibacterial activity

Zones of inhibition of *L. nobilis* extract and standard antibiotics are presented in Table 2. The inhibition zone diameter detected with the extract was 15 mm for *S. aureus*, followed by 14 mm for *E. faecalis* and 8 mm for *K. pneumoniae,* respectively. The antibacterial activity of *L. nobilis* extract was tested at concentrations from 7.8 to 1000 μg/mL, and MIC values were determined to be 125, 250, and 1000 μg/mL for *S. aureus*, *E. faecalis,* and *K. pneumoniae*, respectively.

**Table 2:** Detection of inhibition zones in bacteria.

Microorganisms	Inhibition zones (mm)						
	Positive controls						
	50 μL	GEN	MER	P	CFX	AMP	VAN
*Staphylococcus aureus*	15	NT	NT	18	22	NT	NT
*Enterococcus faecalis*	14	NT	NT	NT	NT	17	13
*Klebsiella pneumoniae*	8	23	31	NT	NT	NT	NT

50 μL: *Laurus nobilis* extract; NT: not tested; GEN: gentamicin 10 μg; MER: meropenem 10 μg; P: penicillin 10 μg; CFX: cefoxitin 30 μg; AMP: ampicillin 2 μg; VAN: vancomycin 5 μg.

### Transforming growth factor-β1 expression

**Figure 2:**
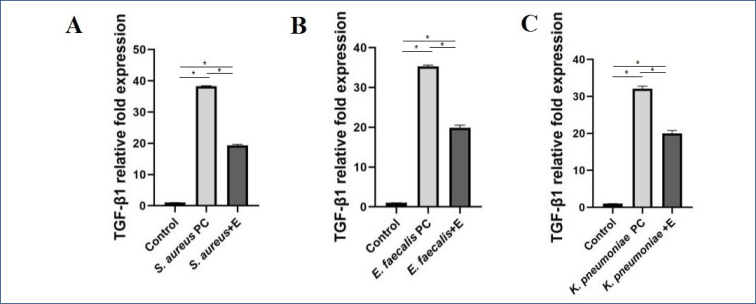
Transforming growth factor-β1expression fold change. (A) *Staphylococcus aureus* TGF-β1 relative fold expression. (B) *Enterococcus faecalis aureus* TGF-β1 relative fold expression. (C) *Klebsiella pneumoniae* TGF-β1 relative fold expression. *p<0.0001.

Positive, negative control and bacteria/extract groups were compared, and the expression results were found to be statistically significant (p<0.0001). The results are shown in Figure 2. Values were presented as means±SD.

## DISCUSSION

One of the global problems is the development of bacterial resistance to antibiotics. People have been using herbal medicine for centuries for its safety, effectiveness, cultural acceptance, and fewer side effects^15^. *L. nobilis* leaves have been used as a medicinal herb and have pharmacological activity that includes antibacterial and anti-inflammatory effect^16^.

The antibacterial effect of laurel essential oil on human pathogenic bacteria was tested by the disk diffusion method against *S. aureus*, *Staphylococcus epidermidis*, *Staphylococcus faecalis*, *Staphylococcus flexneri*, *Pseudomonas aeroginosa, Serratia marcescens, Salmonella Typhi, K. pneuomoniae*, and *Escherichia coli*. The results showed that *L. nobilis* essential oil has potent antibacterial effects^17^. The antibacterial activity of *L. nobilis* essential oil on some bacterial species was determined as follows: *E. coli* (27.1 mm), *E. faecalis* (28.0 mm), and *Salmonella pullorum* (25.2 mm)^18^.

In our study, the inhibition zone diameter detected with the extract was 15 mm for *S. aureus*, followed by 14 mm for *E. faecalis* and 8 mm for *K. pneumoniae*, respectively. These results show that the antibacterial effect of *L. nobilis* essential oil is much stronger than that of the extract. However, the use of extracts for antimicrobial purposes is also effective. In addition, the diameter of the zone of inhibition of the Gram-positive bacteria *S. aureus* and *E. faecalis* is approximately two times larger than that of the Gram-negative bacteria *K. pneuomoniae*. We suggested that this may be a difference due to the uptake of the active ingredients in *L. nobilis* extract into the bacteria depending on the cell walls or membranes of Gram-positive and Gram-negative bacteria. Recent literature information shows that the antimicrobial activity of phenolic compounds is more sensitive in Gram-positive bacteria than in Gram-negative bacteria^19,20^. The differences in the mechanisms of action in Gram-positive and Gram-negative bacteria are also not entirely clear^21^. However, it has been reported that the antimicrobial potential of phenolic compound molecules with hydroxyl groups can result in weaker interactions due to the strong outer membrane electronegativity in the cell wall of Gram-negative bacteria^22^.

In our study, we found that *L. nobilis* extract has an antibacterial effect on *S. aureus> E. faecalis> K. pneumoniae*. Furthermore, we found that epicatechin was present in the highest amount (2113.7 μg/g) in the extract.

In Hep-G2 cells, the IC_50_ values of *L. nobilis* extract were found as follows: ethyl acetate: 3.80 μg/mL, petroleum ether: 10.60 μg/mL, and methanol extract: 23.20 μg/mL^23^.

In this study, we determined the IC_50_ value of *L. nobilis* methanol extract to be 55.17 μg/mL in PBMCs. The changes in the IC_50_ determined in the various studies may be due to the application to different cell cultures. In addition, the environmental conditions under which *L. nobilis* grows can also have an impact.

Traditionally, TGF-β has been suggested to have potent anti-inflammatory effects on the immune system. However, TGF-β can have pro-inflammatory and anti-inflammatory effects depending on the context in which it acts^24^. Our study showed that *L. nobilis* leaf extract altered TGF-β1 expression on PBMCs compared with positive and negative controls. In this study, the results of relative TGF-β1 expression showed a very similar profile for *S. aureus*, *E. faecalis*, and *K. pneumonia.*

The fact that *L. nobilis* leaf extract applied according to the IC_50_ value calculated at a lower dose than the MIC value showed a similar profile in all bacterial/extract groups compared with the controls indicates that the anti-inflammatory effect is largely achieved through inhibition of the TGF-β1 signaling pathway. This demonstrated that *L. nobilis* leaf extract is a candidate for suppressing the inflammatory pathway caused by pathogens due to its anti-inflammatory effect in addition to its antimicrobial activity.

Inflammation is a natural response of the innate and adaptive immune system to infection. However, when inflammation is left uncontrolled, it can lead to autoimmune or autoinflammatory diseases, neurodegenerative diseases, or cancer^25^. *L. nobilis* leaf extract controls inflammation by suppressing activation of the NLRP3 inflammasome^26^.

## CONCLUSION

The leaves of *L. nobilis* contain many phenolic compounds that contribute to the antimicrobial properties of this plant. The extract was also shown to alter TGF-β1 expression on PBMCs. The phenolic compounds are believed to be responsible for these activities. There is a need to expand research by performing more detailed studies and different cell types and in vivo studies.

## References

[1] Gökşen G, Eser E, Ekiz Hİ (2022). Piyasadan temin edilen defne uçucu yağlarinin kalite özelliklerinin karşilaştirilmasi ve antimikrobiyal özelliklerinin belirlenmesi. Çukurova J Agric Food Sci.

[2] Paparella A, Nawade B, Shaltiel-Harpaz L, Ibdah M (2022). A review of the botany, volatile composition, biochemical and molecular aspects, and traditional uses of *Laurus nobilis*. Plants (Basel).

[3] Ford CT, Richardson S, McArdle F, Lotito SB, Crozier A, McArdle A (2016). Identification of (poly)phenol treatments that modulate the release of pro-inflammatory cytokines by human lymphocytes. Br J Nutr.

[4] Oh SA, Li MO (2013). TGF-β: guardian of T cell function. J Immunol.

[5] Algabri SO, Doro BM, Abadi AM, Shiba MA, Salem AH (2018). Bay leaves have antimicrobial and antioxidant activities. J Pathog Res.

[6] Brinza I, Boiangiu RS, Hancianu M, Cioanca O, Erdogan Orhan I, Hritcu L (2021). Bay leaf (*Laurus nobilis* L.) incense improved scopolamine-induced amnesic rats by restoring cholinergic dysfunction and brain antioxidant status. Antioxidants (Basel).

[7] Sancer O, Şahin U, Ateş M, Yünlü S (2022). Evaluation of genotoxic and apoptotic effects of sprouted potato. Potato Res.

[8] Caponio F, Alloggio V, Gomes T (1999). Phenolic compounds of virgin olive oil: influence of paste preparation techniques. Food Chem.

[9] Panda SK, Ravindran B (2013). Isolation of human PBMCs. Bio-protocol.

[10] Panda SK, Ravindran B (2013). In vitro culture of human PBMCs. Bio-protocol.

[11] Sancer O, Öz ZS, Koşar PA (2023). Effect of wheatgrass on human lymphocyte cells. Med J SDU.

[12] Hudzicki J (2009). Kirby-Bauer disk diffusion susceptibility test protocol. ASM.

[13] Fowler PW, Wright C, Spiers H, Zhu T, Baeten EML, Hoosdally SW (2022). A crowd of BashTheBug volunteers reproducibly and accurately measure the minimum inhibitory concentrations of 13 antitubercular drugs from photographs of 96-well broth microdilution plates. eLife.

[14] Tepebaşı MY, Öztürk Ö (2023). miR-21, miR-221, and miR-222 upregulation in lung cancer promotes metastasis by reducing oxidative stress and apoptosis. Rev Assoc Med Bras (1992).

[15] Aslam B, Wang W, Arshad MI, Khurshid M, Muzammil S, Rasool MH (2018). Antibiotic resistance: a rundown of a global crisis. Infect Drug Resist.

[16] Fang F, Sang S, Chen KY, Gosslau A, Ho C-T, Rosen RT (2005). Isolation and identification of cytotoxic compounds from bay leaf (*Laurus nobilis*). Food Chem.

[17] Moghtader M, Farahmand A (2013). Evaluation of the antibacterial effects of essential oil from the leaves of *Laurus nobilis* L. in Kerman Province. J Microbiol Antimicrobials.

[18] Tomar O, Akarca G, Gök V, Ramadan MF (2020). Composition and antibacterial effects of Laurel (*Laurus nobilis* L.) leaves essential oil. J Essent Oil-Bear Plants.

[19] Coman MM, Oancea AM, Verdenelli MC, Cecchini C, Bahrim GE, Orpianesi C (2018). Polyphenol content and in vitro evaluation of antioxidant, antimicrobial and prebiotic properties of red fruit extracts. Eur Food Res Technol.

[20] Wafa BA, Makni M, Ammar S, Khannous L, Hassana AB, Bouaziz M (2017). Antimicrobial effect of the Tunisian Nana variety *Punica granatum* L. extracts against *Salmonella enterica* (serovars Kentucky and Enteritidis) isolated from chicken meat and phenolic composition of its peel extract. Int J Food Microbiol.

[21] Du W, Zhou M, Liu Z, Chen Y, Li R (2018). Inhibition effects of low concentrations of epigallocatechin gallate on the biofilm formation and hemolytic activity of *Listeria monocytogenes*. Food Control.

[22] Rosas-Burgos EC, Burgos-Hernández A, Noguera-Artiaga L, Kačániová M, Hernández-García F, Cárdenas-López JL (2017). Antimicrobial activity of pomegranate peel extracts as affected by cultivar. J Sci Food Agric.

[23] Nagah N, Mostafa I, Osman A, Dora G, El-Sayed Z, Ateya A-M (2021). Bioguided isolation and in-silico analysis of Hep-G2 cytotoxic constituents from *Laurus nobilis* Linn. cultivated in Egypt. Egyptian J Chem.

[24] Villar VH, Subotički T, Đikić D, Mitrović-Ajtić O, Simon F, Santibanez JF (2023). Transforming growth factor-β1 in cancer immunology: opportunities for immunotherapy. Adv Exp Med Biol.

[25] Dinarello CA (2010). Anti-inflammatory agents: present and future. Cell.

[26] Lee EH, Shin JH, Kim SS, Lee H, Yang SR, Seo SR (2019). Laurus nobilis leaf extract controls inflammation by suppressing NLRP3 inflammasome activation. J Cell Physiol.

